# The Truth about Venereal Disease

**Published:** 1921-04-09

**Authors:** Marie C. Stopes

**Affiliations:** Givons Grove, Leatherhead, Surrey


### THE EDITOR'S LETTER-BOX.

**Correspondence on all subjects is invited, but we cannot in any way be responsible for the opinions expressed by our correspondents, who must give their name and address as a guarantee of good faith, but not necessarily for publication. Correspondents are reminded that brevity of style and conciseness of statement greatly facilitate early insertion.**

### "The Truth about Venereal Disease."

*To the Editor of* The Hospital.

Sir,—In your review of my book, "Truth about Venereal Disease," part of which is so cordial, your reviewer says: "It is a great pity that she does not get a medical man to read her proof-sheets." I really think that it is a great pity that a paper of your standing should entrust any book worth reviewing at all to a reviewer who so evidently does not read his books carefully. If you will turn to the first page of my book you will find that it is written at the request and with the approval of Professor W. M. Bayliss, M.A., D.Sc., F.R.S., Sir James Crichton-Browne, M.R.C.S., M.D., F.R.S.E., Dr. Jane Hawthorne, Sir W. Arbuthnot Lane, M.B., M.S., F.R.C.S., and Sir John Macalister, F.S.A., F.R.G.S., so that not only "a medical man," but five of the most distinguished physiologists and medicals in the country have all read and approved the proof-sheets, as well as others whose names are not mentioned. This being so, I do not think it worth while to discuss with your reviewer the points about which he sets up his opinion against mine. Yet it is incumbent upon me to make it clear (as he raises the point that I am not a medical woman) that one whose life is devoted to scientific research and discovery, and who has contributed original discoveries to biological science as well as instructing medical students and doctors, is, by universal consent, granted a higher authority than the ordinary practising medical. So that, when your reviewer says that *because* I do not practise it is "forcing doctors to tell people that Mrs. Stopes does not know what she is talking about" he is merely being funny. Moreover, I am entitled by Act of Parliament to the title of "Doctor," which the majority of medical practitioners are not. I do not know why your reviewer chooses to call me throughout "Mrs. Stopes," instead of Dr. Marie Stopes.—Yours faithfully,

Marie C. Stopes.

Givons Grove, Leatherhead, Surrey,
April 4, 1921.

[1. Does Mrs. Stopes categorically assert that the gentlemen she mentions have read her proof-sheets and approved of their contents? If they did, she omitted to say so in her preface. We should not have thought it possible that Sir Arbuthnot Lane had put this seal of his approval on every statement made in the book; but, if Mrs. Stopes says he did, we must accept the statement. If Mrs. Stopes does not make this assertion, she is being disingenuous.

2. Sir John Macalister is not a physiologist or a medical man.

3. Mrs. Stopes' claims to "higher authority" on medicine than that of the ordinary practising medical man are not likely to receive such "universal consent" as she thinks. It is precisely because she betrays no sense of the value of evidence and no scientific outlook at all that we question this claim. We feel that in botanical science she probably insists on a far higher standard of scientific method and proof than in this other subject; whereas she ought to be stricter in an unfamiliar science than in one in which she is an expert.

4. No statement was made that *because* Mrs. Stopes is not a medical woman she does not know what she is talking about. But that because she writes nonsense now and then medical men have to tell their patients not to put implicit faith in all she says.

5. There is no desire to deprive Mrs. Stopes of her correct title; merely to guard our readers against what we have found to be a universal misconception—namely, that she is a qualified medical woman.

Ed. The Hospital.]

### Should Nurses Smoke?

*To the Editor of* The Hospital.

